# Knockdown of miR-92a suppresses the stemness of colorectal cancer cells via mediating SOCS3

**DOI:** 10.1080/21655979.2021.2022267

**Published:** 2022-02-19

**Authors:** Lifa Li, Jingxiao Zhang, Hong Peng, Xianhong Jiang, Zuoliang Liu, Hongpeng Tian, Songlin Hou, Xingjiang Xie, Qiang Peng, Tong Zhou

**Affiliations:** a Department of Gastrointestinal Surgery II, Hepatobiliary and Pancreatic Research, Affiliated Hospital of North Sichuan Medical College, Nanchong, Sichuan, China; b Department of Medica, The Second Affiliated Hospital of North Sichuan Medical College, Nanchong, Sichuan, China; c Department of Anorectal Surgery, Nanchong Central Hospital, the Second Clinical Medical College, North Sichuan Medical College, Nanchong, Sichuan, China; d Department of Gastrointestinal Surgery II, The Second Affiliated Hospital of North Sichuan Medical College, Sichuan, China; e Department of Gastrointestinal Surgery II, Affiliated Hospital of North Sichuan Medical College, Sichuan, China

**Keywords:** Colorectal cancer, miR-92a, socs3, cancer stem cell, self-renewal, proliferation

## Abstract

MiRNAs (microRNAs) participate in colorectal cancer (CRC) progression and act as potential biomarkers for CRC prognosis. In this study, we investigated the mechanisms of microRNA-92a (miR-92a) in CRC. Expressions of miR-92a and SOCS3 (Suppressor Of Cytokine Signaling 3) were investigated by qRT-PCR in CRC cell lines and 30 cases of CRC. The self-renewal capacity and proliferation of CRC stem cells were estimated by the sphere formation assay, EdU staining, and Flow cytometry analysis. Moreover, the interplay between miR-92a and SOCS3 in CRC cells was validated by luciferase reporter experiments. MiR-92a was found to be remarkably increased while SOCS3 was significantly downregulated in CRC tissues. Inhibition of miR-92a or SOCS3 attenuated the sphere formation capacity, decreased expressions of stemness-related proteins, and inhibited the proliferation of cancer stem-like cells. Knockdown of SOCS3 reversed the repressive impacts of miR-92a inhibitors on self-renewal and growth of CRC cancer stem cells. This study suggested that miR-92a functions as an oncogene of CRC through mediating the stemness of colorectal cancer cells by directly binding and repressing SOCS3.

**Abbreviations:**

CRC, cancer cell; CSCs, cancer stem cells; SOCS3, suppressor of cytokine signaling 3.

## Introduction

Colorectal cancer (CRC), also termed bowel cancer, is a type of tumor that arises from the colon or rectum [1]. The number of CRC patients has continued to rise in recent years, and currently it ranks as the fourth most frequent tumor type in the United States and as the second most fatal malignancy [2]. In 2019, estimated 776,120 males and 768,650 females will be diagnosed as CRC in the United States [3]. Accumulating shreds of evidence have documented that cancer stem cells (CSCs), a subset of cancer cells with strong self-renewal and multiline-differentiation abilities, might act as an important regulator in tumor initiation, development, metastasis, relapse, and drug resistance. Therefore, CSCs are considered to be one of the major hurdles for tumor eradication [4,5]. Understanding the molecular mechanisms and signal cascades initiating CSCs will contribute to developing more effective diagnostic and therapeutic strategies for clinical treatment. Compelling evidence has established that CSCs play a key role during the tumorigenesis of CRC [6]. Nevertheless, the critical factors that determine the acquisition and maintenance of CRC CSCs characters remain unclear.

MicroRNAs (miRNAs), as a type of small non-coding RNAs, are widely presented in mammal cells [7]. Studies reported that miRNAs are closely associated with multiple critical biological processes, such as epigenetic molecular regulation, DNA repair, cell proliferation, apoptosis, and signal transduction [8]. Abnormal expressions of miRNA lead to the destruction of normal physiological activities in mammal cells, resulting in various human diseases, including neurodegenerative diseases, metabolic diseases, autoimmune diseases, and cancers [9,10]. The implication of miRNAs in CRC has been well established by numerous studies showing that miRNAs can serve as both oncogene and tumor repressors of CRC through diverse mechanisms [11,12]. In recent years, miRNAs have been reported to affect CRC tumor growth by regulating the property of CRC CSCs, implying that miRNAs might be a potential regulator of CRC stemness acquisition and maintenance [13]. MiR-92a is a critical miRNA that has been correlated with multiple cancer types, such as esophageal squamous cell cancer, hepatocellular carcinoma, and glioma [14–16]. MiR-92a was recently reported by Peng Q et al. as a promising molecule in predicting risk, recurrence, and survival of CRC [17]. However, the function of miR-92a in CRC stemness has not been determined.

SOCS3 (suppressor of cytokine signaling 3), a critical subtype of the SOCS family, is a negative modulatory agent in growth factors and cytokines-related signaling cascades [18,19]. SOCS3 can repress the JAK-STAT pathway, inhibit cancer cell malignant transformation, and facilitate cancer cell apoptosis [20]. Thus, activation of SOCS3 has been considered a promising therapeutic treatment of tumors [21]. The role of SOCS3 in CRC was demonstrated by previous studies showing that SOCS3 overexpression represses CRC cell proliferation and invasion while facilitating CRC cell apoptosis [22]. The interaction between SOCS3 and miRNAs plays a critical role in CRC tumor progression [23].

The aim of this study was to investigate whether miR-92a and SOCS3 play a role in the stemness of CRC cells. We hypothesized that miR-92a promotes stem-like property of CRC by targeting SOCS3, and thus deteriorates progress of CRC. We also investigated the biological functions of miR-92a and SOCS3 in CRC stemness in vitro, attempting to further explore the mechanisms of CRC.

## Materials and methods

### CRC tumor collection and cell culture

Thirty CRC tissue and matched non-tumor tissues were obtained from patients who were diagnosed at the Second Affiliated Hospital of North Sichuan Medical College as CRC and without chemotherapy. Specimens were kept at −80°C until the study began. Every subject received a written informed consent before this study. This study was approved by the Ethics Committee of the Second Affiliated Hospital of North Sichuan Medical College. Four human CRC cell lines (LoVo, SW480, HT29, and HCT116) and one normal colon epithelial cell line (FHC) were offered by the Chinese Academy of Science cell bank (Shanghai, China). Cells were maintained in RPMI-1640 medium (Invitrogen, USA) supplemented with 10% fetal bovine serum (Gibco, USA).

### Quantitative real-time PCR (qRT-PCR) assay

Total RNAs were extracted from CRC tissues and cells using the TRIzol reagent (Invitrogen) according to the manufacturers’ instructions. The quality of RNAs was tested via NanoDro 2000 c (Thermo Scientific, USA). Subsequently, 3 μg RNAs were utilized as templates to produce cDNA using PrimeScript RT Reagent (Takara, Dalian, China). The SOCS3 mRNA and miR-92a were detected by a SYBR Premix Taq (Takara). The PCR processes were completed on an ABI 7300 Real-Time system (Ambion, USA) with parameters of 95°C for 40 s, 40 cycles of 95°C for 25 s, 58°C for 40 s, and 72°C for 55 s. Primers are shown in Table 1.

**Table 1.** The sequences of primers for Realtime PCR

GENE	Primers	
	Forward (5ʹ-3ʹ)	Reverse (5ʹ-3ʹ)
GAPDH	TGTTCGTCATGGGTGTGAAC	ATGGCATGGACTGTGGTCAT
MiR-92a	ACACTCCAGCTGGGTATTGCACTTGTCCCGG	CTCAACTGGTGTCGTGGAGTCGGCAATTCAGTTGAGACAGGCCG
SOCS3	CTCTGACTCTACACTCGCC	TAGTCCCGAAGCGAAATCTC
CD133	AGTCGGAAACTGGCAGATAGC	GGTAGTGTTGTACTGGGCCAAT
Sox2	GCCGAGTGGAAACTTTTGTCG	GGCAGCGTGTACTTATCCTTCT
OCT4	GGGAGATTGATAACTGGTGTGTT	GTGTATATCCCAGGGTGATCCTC
All R	CTCAACTGGTGTCGTGGA	
U6	CTCGCTTCG GCAGCACA	AACGCTTCACGAATTTGCGT

### Western blot assay

Total proteins were extracted from CRC tissue and cell samples by the RIPA reagent obtained from Sigma, USA, and separated using 10% SDS-PAGE. The isolated proteins were transferred into PVDF membranes followed by 5% nonfat milk incubation to minimize the noise. Next, the membranes were immersed in milk containing primary antibodies against SOCS3 (Rabbit, 1:2000, ab16030, Abcam, UK), CD133 (Rabbit, 1:1000, ab216323, Abcam), SOX2 (Mouse, 1:2000, ab171380, Abcam), OCT4 (Rabbit, 1:5000, ab109183, Abcam), AKT (Rabbit, 1:500, ab8805, Abcam), STAT3 (Mouse, 1:5000, ab119352, Abcam), p-AKT (Rabbit, 1:1000, ab38449, Abcam), p-STAT3 (Rabbit, 1:5000, ab76315, Abcam), and GAPDH (Rabbit, 1:3000, ab124905, Abcam), and incubated overnight. After excess primary antibodies were washed off by PBS, the membranes were probed by HRP-conjunct secondary antibodies, and the signals were visualized using the ECL reagent (Bio-Rad).

### RNA transfection

The miR-92a inhibitors, pcDNA3.0-SOCS3, SOCS3 siRNA, and their corresponding controls, including the miR-92a negative control and SOCS3 negative control, were provided by GenePharma (Shanghai, China). For cell transfection, CRC cells (2 × 10^6^ cells/well) were seeded into 96-well plates and cultured at 37°C for 10 h. Cells were then transfected with the indicated RNAs using Lipofectamine 3000 (Invitrogen).

### CSC cell culture and sphere formation analysis

CRC cancer stem-like cells (CSCs) were enriched using a serum-free culture medium. CRC CSCs were maintained in ultra-low attachment flasks (Corning, MA, USA) as spheres in RPMI-1640 containing nonessential amino acids (Fisher, USA), penicillin–streptomycin (Fisher), B27 supplement (Invitrogen, USA), and epidermal growth factor (50 ng/mL, Invitrogen). For sphere formation detection, CSCs (1,000 cells) were seeded into 6-well ultra-low attachment plates (Corning), and tumor spheres were counted after 2 weeks of culture using a microscope (Olympus).

### EdU staining

**The** Cell-Light™ EdU detection Kit (Ribobio) was used to examine cell proliferation. In brief, CRC cells were plated into poly-L-lysine coated 96-well plates and maintained at 37°C for 8 h. Next, EdU (50 μM) was added to each well and allowed to incubate with CSC cells for an additional 24 h, followed by fixation of 4% paraformaldehyde. Finally, the treated cells were observed by a DMR fluorescent microscope (Leica, Germany).

### Luciferase reporter assay

To validate the interplay between SOCS3 and miR-92a, a luciferase reporter assay was conducted on CRC cells. Briefly, the wild-type miR-92a target region of SOCS3 3ʹ-UTR was cloned into psi-CHECK2 (Origene, Rockville, MD) to establish a luciferase reporter plasmid, SOCS3-WT. The mutant target region was also inserted into psi-CHECK2 to construct SOCS3-Mut. The SOCS3-WT or SOCS3-Mut was co-transfected into CRC cells with miR-92a mimics or inhibitors using Lipofectamine 3000 (Invitrogen). The change of luciferase activity was monitored using a Dual-Luciferase Assay System (Promega) kit under the guidance of the manufacturer.

### Statistical analysis

All data are shown as mean ± SEM, and one-way ANOVA analysis, or student’s t test, was conducted on the GraphPad Prism (Version 7.0, USA) to estimate the difference between groups. P < 0.05 was regarded as statistically significant.

## Results

*In this study, we first detected the differential expression of SOCS3 in cancer tissues and para-cancerous tissues, and then detected the expression level of miR-92a in tumor cell lines. Then, in the colorectal cancer cell line, miR-92a was knocked down, and then the cell sphere formation, the expression level of CD133 et al. and cell proliferation activity were detected. Then, the 3ʹUTR region in which miR-92a can target SOCS3 was detected by dual-Luciferase report assay, and the inhibitory effect of miR-92a on the expression of SOCS3 was determined by Western blot. In colorectal cancer cell line, SOCS3 was over-expressed, and the cell sphere formation, the expression level of CD133 et al. and cell proliferation activity were detected. Finally, knockdown of miR-92a and SOCS3, cell sphere formation, expression level of CD133 et al. and cell proliferation activity of colorectal cancer cells were detected. Experiments above elucidate the effect of miR-92a/SOCS3 on the malignant behavior of colorectal cancer cells.*

### SOCS3 was lowly expressed while miR-92a was highly expressed in CRC

**Figure 1.**
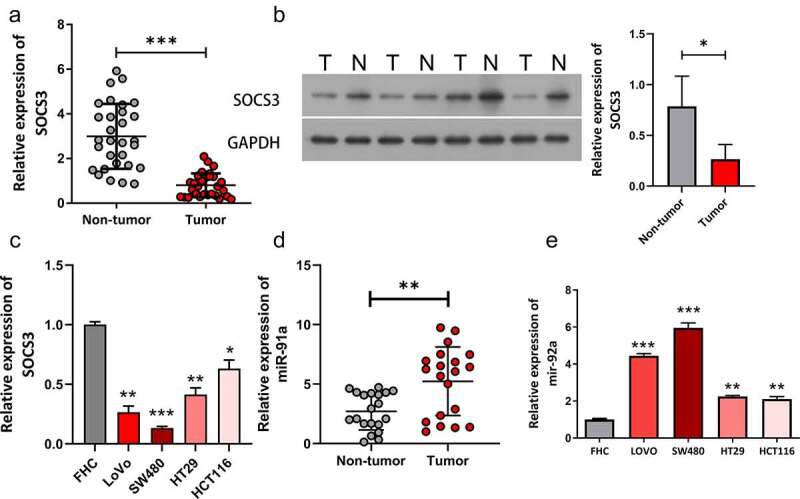
**SOCS3 was lowly expressed while miR-92a was highly expressed in CRC**. (a) **The** SOCS3 mRNA level was examined by qRT-PCR in CRC and matched normal samples, ***P < 0.001 vs non-tumor samples. (b) Expression of the SOCS3 protein was measured using Western blot in CRC and matched normal samples, *P < 0.05 vs non-tumor samples. (c) Expression of SOCS3 was measured using qRT-PCR in FHC and CRC cell lines. * P < 0.05, **P < 0.01, ***P < 0.001 vs FHC group. (d) Expression of miR-92a was measured using qRT-PCR in clinical tissues. (e) The relative levels of miR-92a in four CRC cell lines and FHC cells were detected by qRT-PCR, **P < 0.01, ***P < 0.001 vs FHC group.

To explore the role of SOCS3 and miR-92a during CRC tumorigenesis, we first examined expressions of SOCS3 in 30 CRC clinical tissues and cell lines. Results obtained from qRT-PCR showed that SOCS3 mRNA was remarkably decreased in CRC tissue samples compared with matched normal samples (Figure 1a, ***P < 0.001). Moreover, protein expression of SOCS3 was also downregulated in CRC tumor samples compared to that in non-tumor samples (Figure 1b). Results of qRT-PCR showed that expression of SOCS3 in cancer cell lines was lower than that in FHC cells (Figure 1c). Further experiments presented that miR-92a expression was higher in tumor tissues than non-tumor tissues (Figure 1d). In addition, miR-92a was upregulated in CRC cell lines (LoVo, SW480, HT29, and HCT116) compared to the FHC cell line (Figure 1e, **P < 0.01, ***P < 0.001). We also found that miR-92a expression in CRC cell lines, LoVo and SW480 was higher than that in HT29 and HCT116. Therefore, CRC cell lines, LoVo and SW480 were used in subsequent experiments.

### Inhibition of miR-92a repressed self-renewal and growth of CSC cells

**Figure 2.**
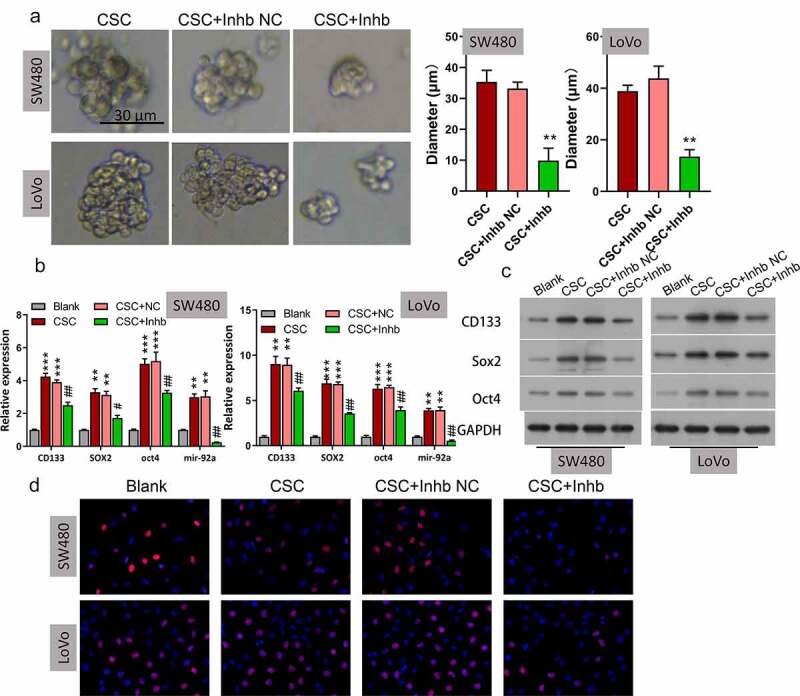
**Inhibition of miR-92a repressed self-renewal and growth of CSC cells**. (a) **A** serial sphere propagation experiment was used to estimate the influence of miR-92a knockdown on CSC self-renewal, **P < 0.01 vs CSC+NC. (b) Levels of CD133, Sox2, and Oct4 were evaluated by qRT-PCR in SW480 and LoVo cells, **P < 0.01, ***P < 0.001 vs blank; ## P < 0.01 vs CSC+NC. (c) Protein expressions of CD133, SOX2, and OCT4 were detected in SW480 and LoVo cells. (d) EdU staining was used to assess the effects of miR-92a knockdown on CSC cell proliferation.

**Abbreviations**: CSC, cancer stem cells.

Due to that tumor stemness has been closely correlated to tumor initiation, migration, recurrence, and even drug resistance, we then investigated the effect of miR-92a on the stemness-related properties of CRC CSC cells, including the self-renewal capacity and levels of CRC CSC markers. By using two CRC CSC cell lines (SW480 and LoVo) treated with the miR-92a inhibitor or matched inhibitor NC, we found that miR-92a inhibition markedly repressed the sphere formation ability (Figure 2a, **P < 0.01). To further confirm this finding, qRT-PCR and Western blot analysis were used to estimate expressions of stemness-related proteins in SW480 and LoVo CSC cells. Results indicated that CD133, SOX2, and OCT4 were all significantly elevated in CSC cells than in non-CSC CRC cells, and knockdown of miR-92a remarkably decreased their expression in CSC cells (Figure 2b and 2c, **P < 0.01, ***P < 0.001; ## P < 0.01). We performed EdU staining to evaluate the role of miR-92a inhibition on CRC CSCs growth. Results from EdU staining revealed that miR-92a inhibition resulted in significant repression of cell growth (Figure 2d). These results demonstrated that miR-92a inhibition remarkably suppressed self-renewal and growth of CSC cells.

### SOCS3 was bound and negatively modulated by miR-92a in CSC

**Figure 3.**
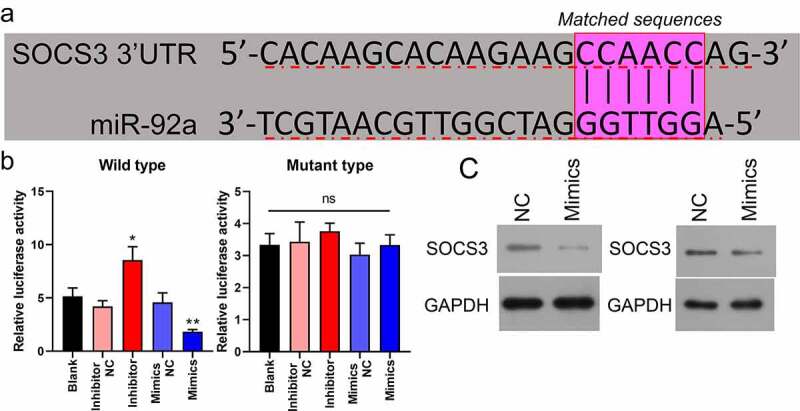
**SOCS3 was bound and negatively modulated by miR-92a in CSC**. (a) The putative binding site sequence between miR-92a and SOC3C 3ʹ-UTR. (b) Interplay between miR-92a and SOCS3 was validated by luciferase reporter experiments, *P < 0.05 vs inhibitor NC, *P < 0.01 vs mimics NC. (c) Western blot analysis of SOCS3 in miR-92a silenced SW480 and LoVo cells.

By using bioinformatics analysis, it was found that the 3ʹ-UTR regions of SOCS3 possessed a miR-92a binding sequence (Figure 3a). We investigated whether there is an interaction between miR-92a and SOCS3 in CRC through dual-luciferase reporter experiments. The luciferase activities of CRC cells driven by the wild-type SOCS3 plasmid could be markedly enhanced or attenuated by the miR-92a inhibitor or miR-92a mimics, respectively. In contrast, the luciferase activities of those cells driven by the mutant type of the SOCS3 plasmid could not be affected by the miR-92 inhibitor or mimics (Figure 3b, *P < 0.05, **P < 0.01). In addition, using Western blot, it was demonstrated that miR-92a overexpression resulted in a remarkable downregulation of SOCS3 in SW480 and LoVo cells (Figure 3c). These findings indicated that SOCS3 was bound and negatively modulated by miR-92a.

### Overexpression of SOCS3 repressed self-renewal and growth of CSC cells

**Figure 4.**
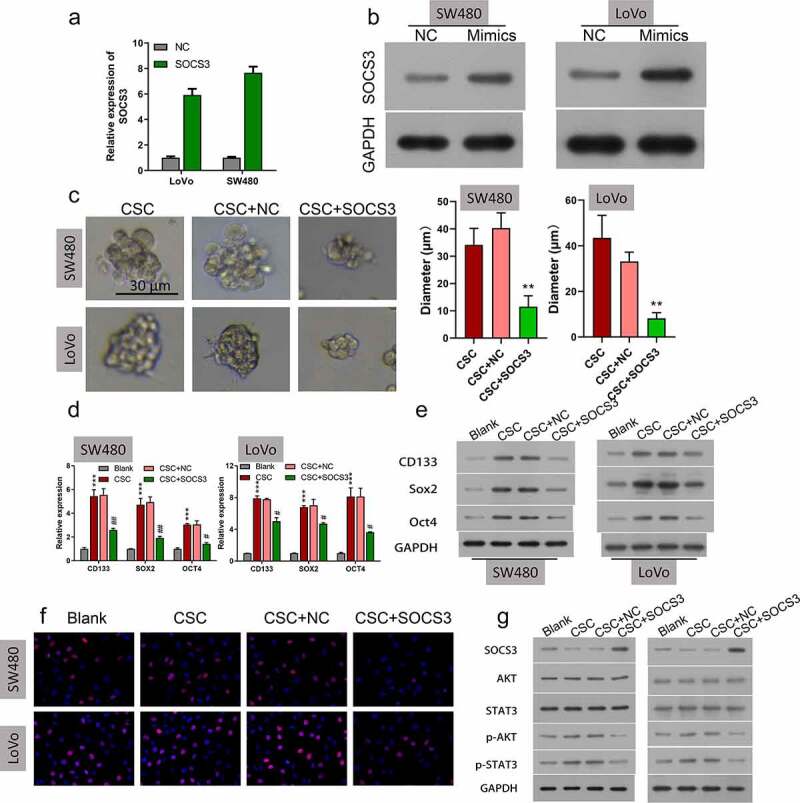
**Overexpression of SOCS3 repressed self-renewal and growth of CSC cells**. (a and b) qRT-PCR and Western blot were used to examine the SOCS3 overexpression efficiency in SW480 and LoVo cells, ***P < 0.001 vs NC. (c) Influences of SOCS3 overexpression on CSC self-renewal were estimated by the sphere propagation assay in SW480 and LoVo cells, **P < 0.01 vs CSC+NC. (d) qRT-PCR and (e) Western blot were carried out in SOCS3 overexpressed SW480 and LoVo CSC cells to examine the protein levels of CD133, SOX2, and OXT4, ***P < 0.001 vs blank; ##P < 0.01 vs CSC+NC. (f) Cell proliferation was evaluated by EdU staining in SW480 and LoVo CSC. (g) Protein expressions of SOCS3, AKT/p-AKT, and STAT3/p-STAT3 in SOCS3 overexpressed SW480 and LoVo CSC cells were assessed via Western blot.

To understand whether SOCS3 mediates self-renewal and growth of CRC in CSC cells, SOCS3 was overexpressed in SW480 and LoVo CSC cells, and then the overexpression efficiency of SOCS3 was examined. Results indicated that SOCS3 was significantly increased in SOCS3 transfected SW480 and LoVo CSC cells (Figure 4a-b). In the sphere formation analysis, it was found that SOCS3 overexpression markedly attenuated the sphere formation capacity of SW480 and LoVo CSC cells (Figure 4c, **P < 0.01). Also, it was found that CD133, SOX2, and OCT4 expression was downregulated in SOCS3 transfected SW480 and LoVo CSC cells compared to negative control groups (Figure 4d-e, ***P < 0.001, #P < 0.05). Moreover, results obtained from EdU staining indicated that SOCS3 overexpression caused a significant repression of cell proliferation of SW480 and LoVo CSC cells (figure 4f, ***P < 0.001). In addition, we found that the protein levels of p-AKT and p-STAT3 were remarkably downregulated in SOCS3 treated SW480 and LoVo CSC cells compared to negative control cells (Figure 4g). These results demonstrated that overexpression of SOCS3 repressed self-renewal and growth of CSC cells.

### Knockdown of SOCS3 reversed the repressive effects of the miR-92a inhibitor on self-renewal and growth of CSC cells

**Figure 5.**
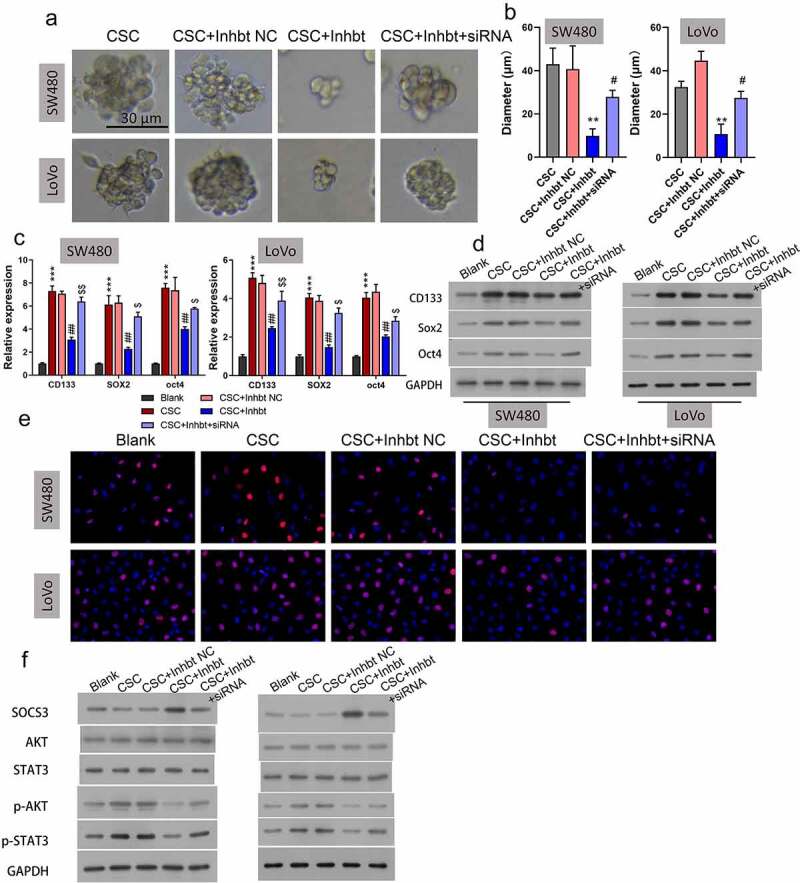
**Knockdown of SOCS3 reduced the repressive functions of the miR-92a inhibitor on self-renewal and growth of CSC cells**. (a and b) Representative images and statistic results of CSC spheres with treatment of the miR-92a inhibitor and miR-92a + SOCS3 siRNA, **P < 0.01 vs CSC+NC group; #P < 0.05 vs CSC+miR-92a inhibitor. (c) qRT-PCR and (d) Western blot were utilized to estimate the relative expressions of CD133, SOX2, and OCT4 on the miR-92a inhibitor and miR-92a + SOCS3 siRNA treated SW480 and LoVo CSC cells. (f) Cell growth of different groups (Blank, CSC, CSC+inhibitor NC, CSC+miR-92a inhibitor, and CSC+miR-92a inhibitor+SOCS3 siRNA) were detected via EdU staining. (g) Western blot detection of SOCS3, AKT, STAT3, p-AKT, and p-STAT3 in different groups (Blank, CSC, CSC+inhibitor NC, CSC+miR-92a inhibitor, and CSC+miR-92a inhibitor+SOCS3 siRNA).

Because miR-92a inhibition and SOCS3 overexpression were both demonstrated to repress the self-renewal and growth of CRC CSC cells, and miR-92a could bind and negatively regulate SOSC3, we speculated that SOCS3 might be involved in the tumorigenesis of CRC by acting downstream of miR-92a. To validate this speculation, we treated miR-92a silenced SW480 and LoVo CSC cells with SOCS siRNA, followed by the detection of cell self-renewal and growth. Results illustrated that treatment of SOSC3 siRNA could remarkably reverse the repression of sphere formation ability induced by the miR-92a inhibitor (Figure 5a-b, **P < 0.01, #P < 0.05). Correspondingly, the miR-92a inhibitor caused downregulation of CD133, SOX2, and OCT4 that was also demonstrated to be abolished by SOCS3 siRNA treatment in SW480 and LoVo CSC cells at both mRNA (Figure 5c, ***P < 0.001, ##P < 0.01) and protein (Figure 5d) levels. We also demonstrated that the repression of cell proliferation of SW480 and LoVo CSC cells caused by miR-92a inhibitor transfection could be reversed by SOCS3 siRNA (Figure 5e). In addition, the reduction of p-AKT and p-STAT3 in miR-92a silenced SW480 and LoVo CSC cells were also abrogated by the SOCS3 siRNA treatment (figure 5f). These findings indicated that knockdown of SOCS3 reversed the repressive effects of the miR-92a inhibitor on self-renewal and growth of CSC cells.

## Discussion

In this study, miR-92a and SOCS3 were revealed to be upregulated and downregulated. In the functional analysis, miR-92a inhibition and SOCS3 overexpression were both demonstrated to attenuate the CRC CSC cell self-renewal capacity, and repressed CRC CSC cell proliferation. MiR-92a was revealed to bind and negatively modulate SOCS3 in CRC cells. Moreover, SOCS3 knockdown could abolish the suppressive impacts of the miR-92a inhibitor on the self-renewal capacity of CRC CSC cells. Hence, miR-92a facilitates the stemness of CRC cells by inhibiting SOCS3, suggesting that the miR-92a/SOCS3 axis might be a promising therapeutic target of CRC.

The existence of CSC cells and their profiles has been well documented by more and more literature referring to the initiation, progression, and recurrence of CRC [24]. As reported, CSC cells are a subset of cancer cells, characterized by immature biomarkers, self-renewing, and strong drug-resistance [25]. CSC cells can originate from non-stem, non-differentiated, or chemically treated tumor cells [26]. The drug-resistance ability of cancer cells with significantly increased expression of stemness proteins, such as CD133, SOX2, and OCT4, is much stronger than those cells with either low or no stemness markers [27,28]. However, CRC stemness and its underlying regulatory mechanisms remains largely undetermined. miRNAs have important impacts on the stemness of various of human cancer cells [29]. For example, miR-206 was reported to repress stemness and metastasis of breast tumor cells by affecting the MKL1/IL-11 cascade [30]. Moreover, Guo JC et al. found that miR-448 can attenuate the self-renewing of HCC (hepatocellular carcinoma) stem cells by suppressing stemness maintenance, and the MAGEA6-mediated AMPK cascade was demonstrated to contribute to this phenomenon [31]. Similarly, in CRC, miR-196a was reported to enhance the stemness of CRC cells by repressing ZG16 [32]. MiR-92a was previously found to facilitate the tumorigenesis of CRC [33,34]. Nevertheless, whether miR-92a has an impact on CRC stemness remained uninvestigated. Here, we provided solid evidences that inhibition of miR-92a can destroy the stemness of CRC cells.

The JAK-STAT cascade was demonstrated to be a regulator of cytokine signaling [35]. Inflammatory agents, including IL-1β, IL-6, and IFN-γ, play a key role in DNA methylation, and thereby have a close association with cancer, and the JAK-STAT cascade is a bridge between inflammation and cancer [36]. Suppressor of cytokine signaling (SOCS) proteins could negatively regulate cytokine signaling mediated by the JAK-STAT cascade via a classical feedback loop [21]. In particular, SOCS1 [37,38] and SOCS3 [39], as potent inhibitors of JAKs, were reported to be played a critical role in various malignant processes, such as in cancers. SOCS1 suppressed metastatic progression of colorectal tumors by preventing the mesenchymal–epithelial transition (MET) [40]. Moreover, Letellier et al [41]. reported that SOCS2 and SOCS6 may be biomarkers in CRC. Knockdown of SOCS3 activates multiple oncogenes, resulting in the emergence of tumors [42]. Low SOCS3 gene expression was reported to be associated with the metastasis of CRC cells [43]. A study performed by Jablons DM et al. discovered that inhibition of SOCS3 by hypermethylation significantly facilitated the tumor growth of lung cancer [44] and ulcerative colitis-related colorectal cancer [45], suggesting that hypermethylation might be the predominate reason responsible for the downregulation of the SOCS3 protein, which further contributes to cancer progression by activating of the STAT3 cascade. Nevertheless, the mechanisms that modulate SOCS3, and the reason of the dysregulation of the JAK/STAT signaling cascade in CRC cells, remain undetermined. Recently, miR-196b-5p was found to facilitate stemness and resistance of CRC cells by targeting SOCS1 and SOCS3, leading to the activation of the STAT3 cascade [23]. This finding suggested to us that the interaction between miRNAs and SOCS3 might be one of the main mechanisms underlying CRC stemness acquisition and maintenance. Here, we found that SOCS3 was dysregulated in CRC, and overexpression attenuated the CRC stemness. Moreover, SOCS3 can be bound by miR-92a, and knockdown of SOCS3 can abrogate the impacts of the miR-92a inhibitor on CRC stemness.

## Conclusion

Our study suggested that miR-92a might have a critical role in the stemness of CRC cells by repressing SOCS3, improving our knowledge of CRC cell proliferation and stemness, and this result will contribute to developing effective therapeutic measures against CRC. As far as we know, this study is the first to report the exploration of the mechanism of miR-92a in the maintenance of stem-like property in colorectal cancer, which will provide more complete information for the involvement of miR-92a in the metastasis of colorectal cancer and lay a certain foundation for the pathogenesis of colorectal cancer.
